# A Rare Case of Multiple Bilateral Lymphoepithelial Cysts of Palatine Tonsils

**DOI:** 10.7759/cureus.76797

**Published:** 2025-01-02

**Authors:** Dhruvin Shah, Ajeet K Khilnani, Narendra Hirani, Rashmi Sorathiya, Parth Pomal

**Affiliations:** 1 Otolaryngology-Head and Neck Surgery, Gujarat Adani Institute of Medical Sciences, Bhuj, IND

**Keywords:** benign lymphoepithelial cyst, branchial cyst, palatine tonsils, tonsillar cyst, tonsillectomy

## Abstract

Lymphoepithelial cysts (LECs) are benign developmental cysts of the lymphoid tissue. LEC of palatine tonsils is a rare entity. However, it should be considered in the differential diagnosis of tonsillar enlargement, especially if it is unilateral involvement. The management ranges from conservative observation to surgical resection. Here, we report a case of multiple LECs involving both palatine tonsils for which bilateral tonsillectomy was done. The patient was followed up for six months without any recurrence of similar pathology in the tonsillar region.

## Introduction

Lymphoepithelial cysts (LECs) are benign developmental cysts that develop from lymphoid tissue. LECs can occur in various organs, such as the pancreas [1], thyroid [2], and mediastinum [3]. In the head and neck, they are more common in the lateral neck, also known as a branchial cyst. Oral LECs are smaller than branchial cysts but microscopically identical [4]. Yang X et al. concluded that the most common sites for an intraoral LEC are the tongue and floor of the mouth, which account for 88.3% of cases, and it affects females more than males (2:1) at any age in the case series published of 120 cases of oral LECs [5]. LECs in the palatine tonsils are rare, and very few cases have been reported. Depending on the patient's clinical history, the size and location of the lesion, and their level of discomfort, oral LEC can be treated conservatively or surgically. For example, people with tiny, asymptomatic oral LECs can have periodic monitoring. These small oral LECs have the potential to burst and vanish without surgery. Surgical resection is advised if it is symptomatic, and it has a low risk of recurrence or malignant transformation. As far as we know, there are only seven cases of LECs in palatine tonsils reported so far, out of which there is only one case of bilateral multiple tonsillar LECs. Here, we are reporting a case of multiple LECs in bilateral palatine tonsils.

## Case presentation

**Figure 1:**
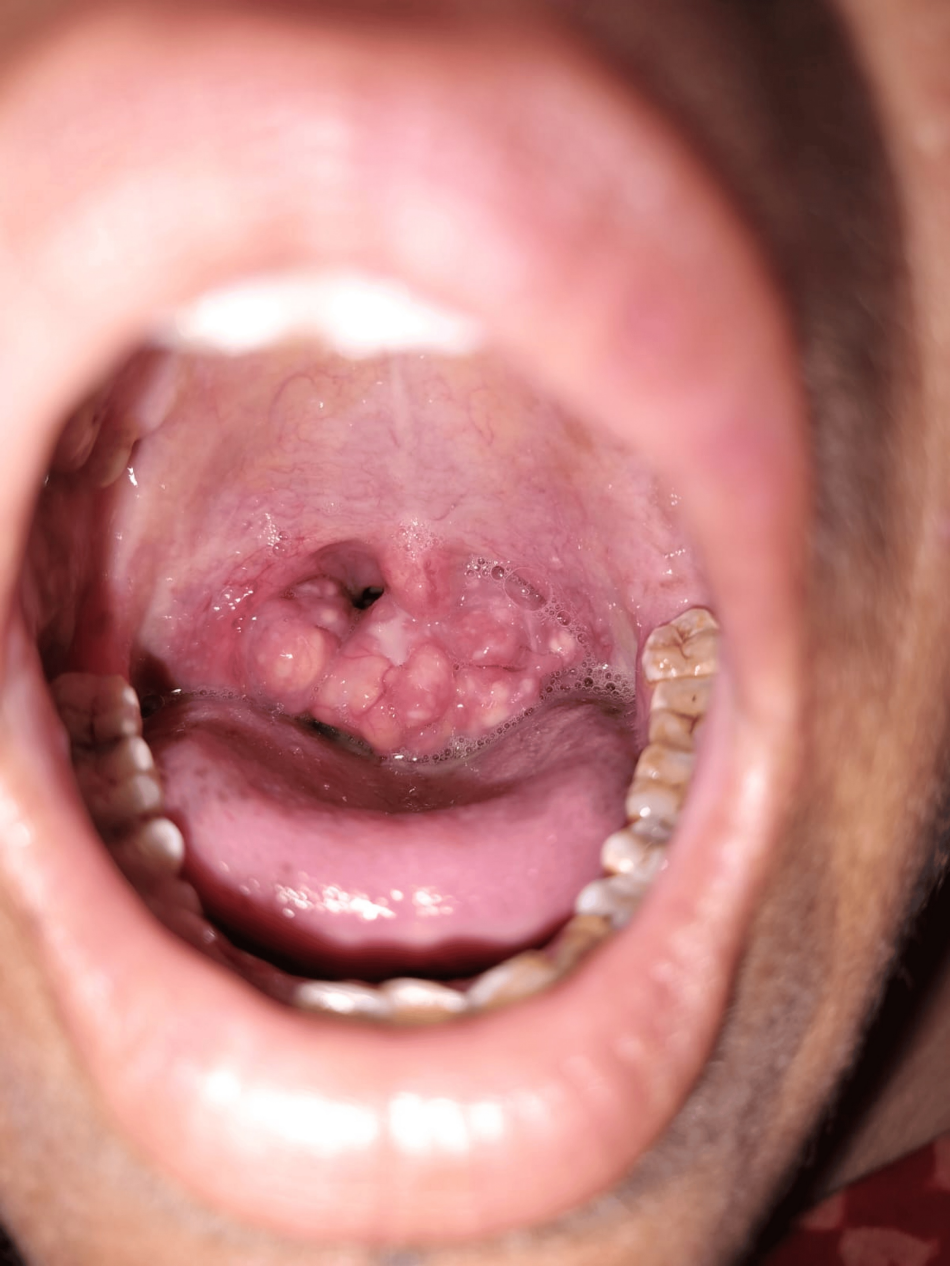
Clinical photograph taken pre-operatively showing left-side grade 4 and right-side grade 3 tonsil with irregular surface obstructing oropharynx

A 37-year-old Hindu female patient visited the ENT outpatient department at GK General Hospital, Bhuj (Gujarat, India), with complaints of occasional pain and difficulty in swallowing for the past three months. The symptoms were insidious in onset, gradually progressive in nature, and occurred more with solid food than liquid. She also mentioned having a lump in her throat, as she had already checked herself in the mirror. There was no history of fever, fatigue, body rashes, stuffy nose, headache, earaches, stiff neck, hoarseness, recurrent sore throat, coughing, sneezing, snoring, or difficulty breathing. She also denied hemoptysis and unexplained weight loss in the last few weeks. Oral examination revealed left grade 4 tonsil and right grade 3 tonsil, obstructing the oropharynx (Figure 1).

**Figure 2:**
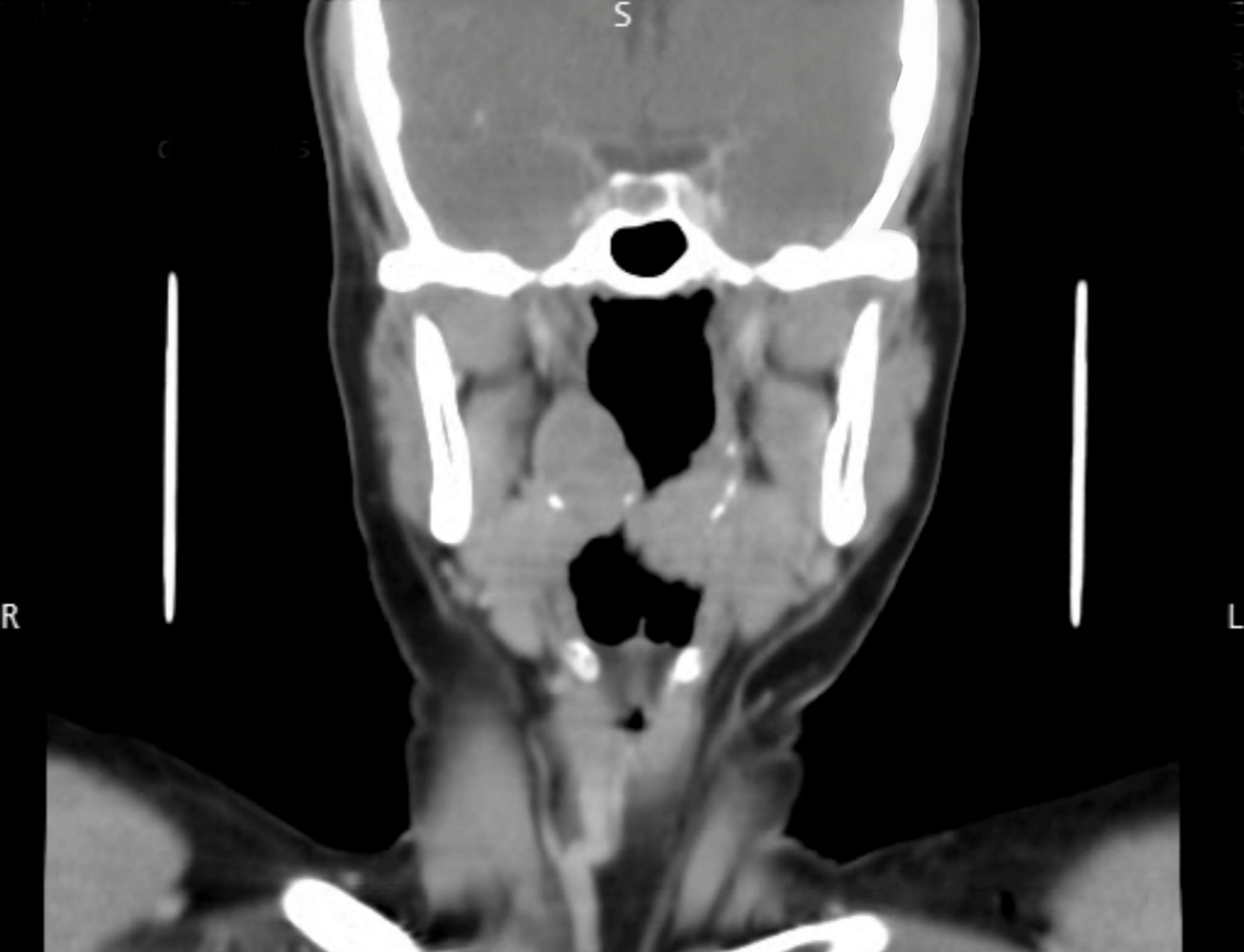
Coronal section of contrast-enhanced CT scan showing bilaterally enlarged tonsils with heterogeneous post-contrast enhancement and foci of peripheral calcification

The extraoral head and neck clinical examination was unremarkable. The patient was advised to undergo a contrast-enhanced CT scan of the neck, which was suggestive of soft tissue density areas present in the bilateral tonsillar region, approximately 23 × 23 mm on the right side and 22 × 31 mm on the left side, with heterogeneous post-contrast enhancement and foci of peripheral calcification (Figure 2).

**Figure 3:**
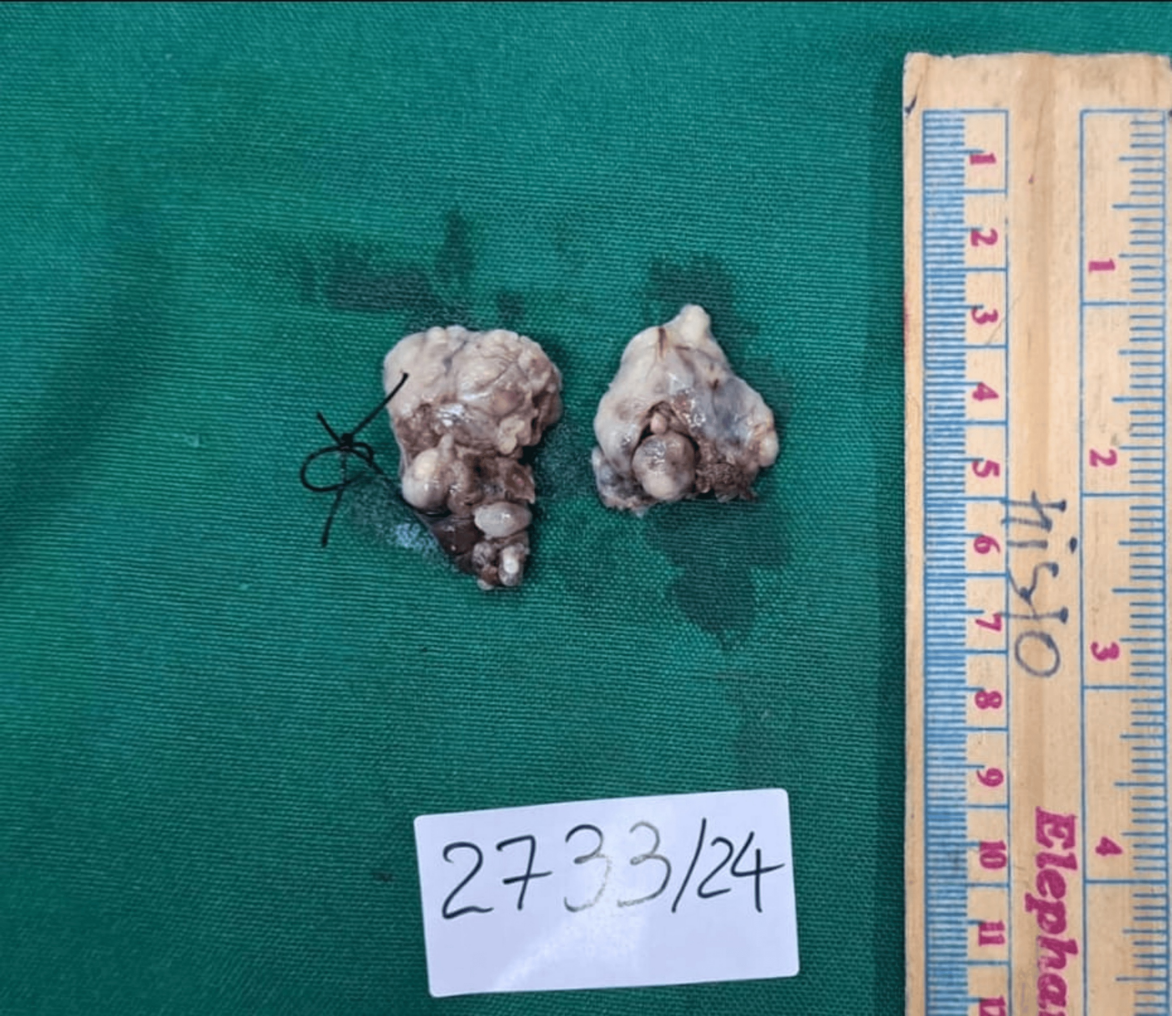
Postoperative specimen of bilateral tonsils showing whitish-brown areas with cysts

All blood reports were within normal limits. Bilateral tonsillectomy by dissection method was done under general anesthesia. Both tonsils were sent for histopathology. On gross examination, the right tonsil measured 3×2.4×1.7 cm, and the left tonsil measured 3.7 × 2.4 × 1.7 cm. Upon sectioning, whitish-brown areas were noted along with multiple cysts (Figure 3).

**Figure 4:**
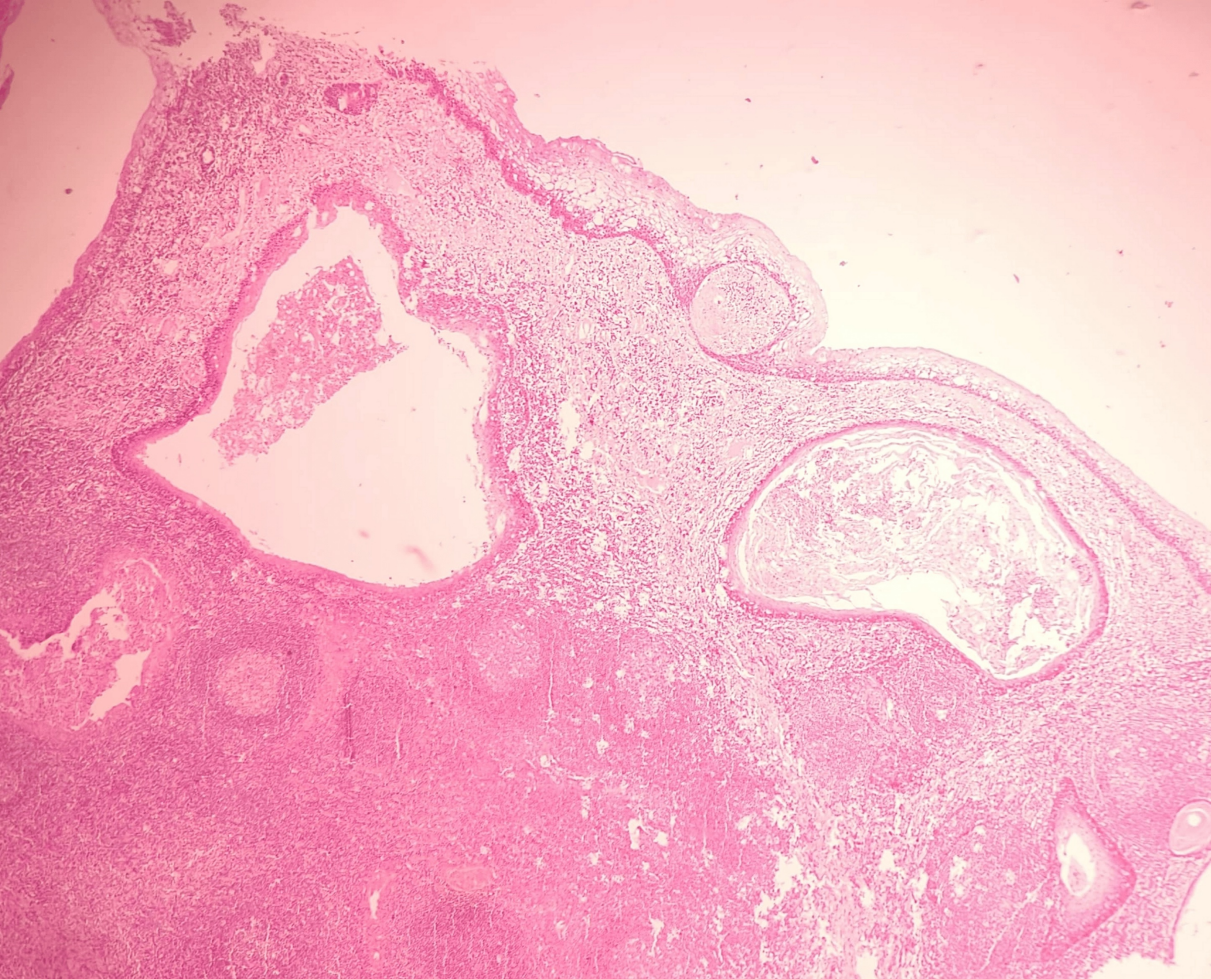
(H & E stain, 10x) Section shows para keratinized stratified squamous epithelium encasing lymphoid follicle of variable size and shape. Two cysts lined by benign stratified squamous epithelium containing loose keratin material also seen

In both tonsils, a few cystic areas were present, lined by parakeratinized stratified squamous epithelium and containing flakes of keratin material and debris. The cyst wall was surrounded by lymphoid cells with occasional germinal centers. No evidence of granuloma or malignancy was found (Figure 4).

**Figure 5:**
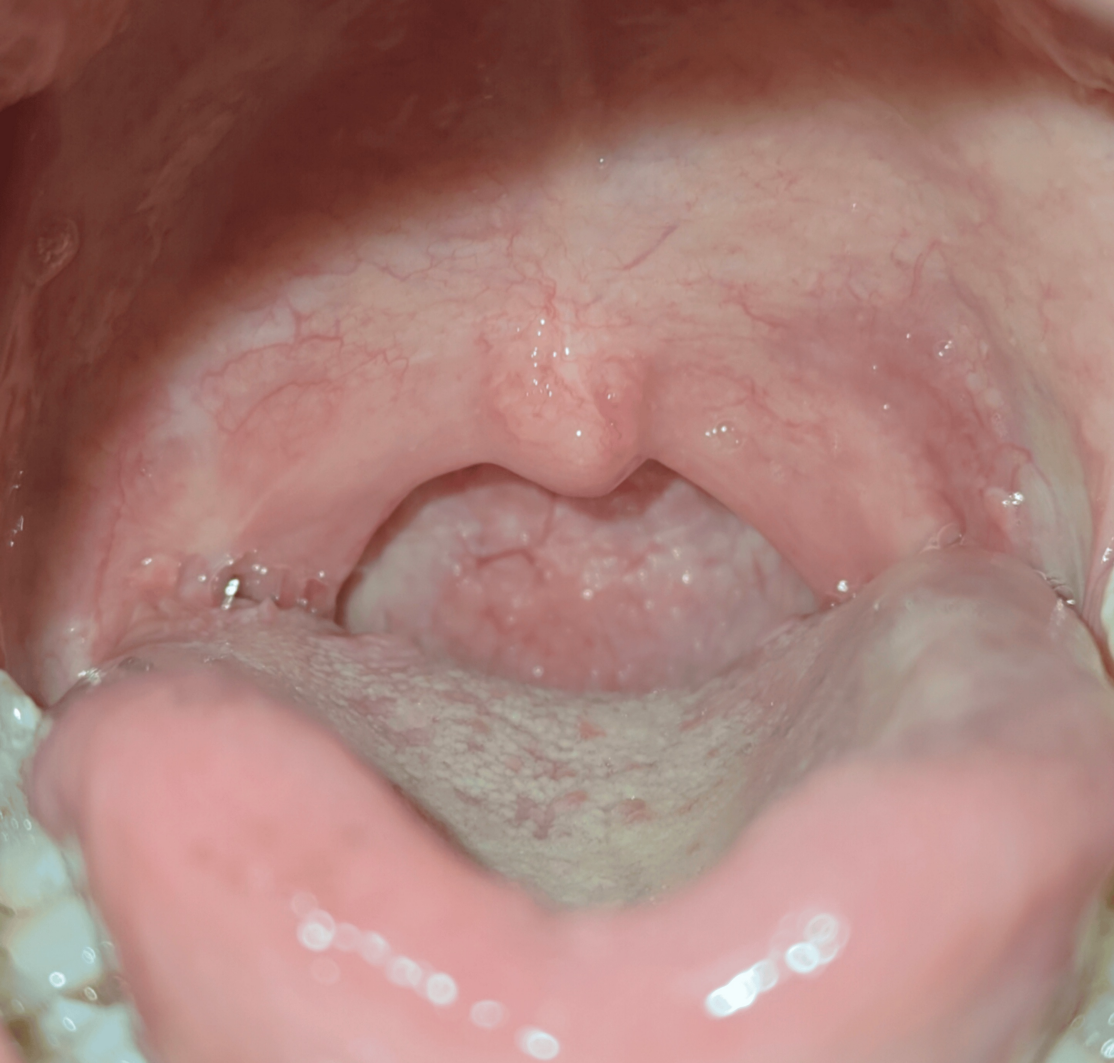
Postoperative clinical photograph taken after six months of surgery

These features were consistent with the diagnosis of lymphoepithelial cysts in bilateral tonsils. There were no significant side effects or recurrences at the six-month follow-up, as shown in Figure 5.

## Discussion

LECs can occur anywhere in the body but are very rare in the oral cavity. According to Giunta J et al., out of all the oral biopsy specimens that were sent for biopsy, only 0.09% were identified as LECs [6]. Since LECs in palatine tonsils are rare, their features, pathogenesis, and management are not widely available. They can be misdiagnosed as lipomas, mucoceles, or dermoid cysts [7]. Numerous ideas have been put forth to explain the development of oral LECs, and the pathophysiology of these conditions remains contentious. Knapp's theory is still the most well-known when it comes to the pathophysiology of oral LECs. According to Knapp, these oral cysts, which are actually pseudo cysts, originate from submucosal lymphoid clusters seen on the tongue, soft palate, and the floor of the mouth, rather than lymph nodes [7]. As stated by Buchner et al. [8] and Giunta J et al. [6], LECs develop when the crypt of the palatine tonsil becomes blocked, creating a dilated cavity that is lined by epithelium containing desquamated cells and keratin. Bhaskar et al. attributed LECs to the proliferation of glandular epithelial cells [9].

The histopathological analysis of oral LECs shows a thick cystic area walled with desquamated keratin and partially keratinized stratified squamous epithelium that is filled with viscous fluid. The cyst wall is penetrated by lymphocytes arranged in germinal centers [8].

As far as the management of LECs in the palatine tonsil is concerned, if there is significant tonsillar hypertrophy and the patient is symptomatic, then surgical resection of the tonsil is advised; otherwise, for small cysts, it can be managed conservatively.

To the best of our knowledge, there are seven reported cases of palatine tonsils LECs in the literature (Table 1).

**Table 1:** Review of the previously reported cases of LECs of palatine tonsil

Author	Report Year	Age	Gender	Location
Tanaka et al. [10]	2004	55	Male	Right tonsil
Kwon et al. [11]	2006	10	Male	Right tonsil
Choi et al. [12]	2010	30	Female	Left tonsil
Mohsenifar Z et al. [13]	2013	72	Male	Bilateral tonsils
Ferreira GM et al. [14]	2015	21	Male	Right tonsil
Balta H et al. [15]	2016	66	Female	Right tonsil
Jae Yeon Moon et al. [16]	2022	37	Female	Left tonsil
Our case report	2024	37	Female	Bilateral tonsils

The data spans nearly two decades, from 2004 to 2024. Out of which six cases were unilateral, and one was bilateral. The complaints of the patients may vary and are associated with the size of the tonsils and cysts; however, difficulty in swallowing is common in patients.

## Conclusions

Lymphoepithelial cysts are rare and lack specific clinical characteristics that would allow for a definitive clinical diagnosis other than through histopathology. LECs in palatine tonsils are uncommon but must be included in the differential diagnosis for unusual tonsillar enlargements, particularly in unilateral instances. Nonetheless, our situation involved bilateral multiple cysts, while most documented cases pertained to unilateral LEC. Surgery is preferred if the patient shows symptoms; otherwise, it can be handled conservatively since it does not have malignant potential.
